# Association of interleukin 6 and uric acid levels in patients with type 2 diabetes mellitus

**DOI:** 10.6026/973206300181136

**Published:** 2022-12-31

**Authors:** Setty Raghav Gutlur Nagarajaiah, Pallavi Somashekar, Rangaswamaiah Hareesh, Ganesh Veluri

**Affiliations:** 1 Department of General Medicine, Rajarajeswari Medical College and Hospital, Bangalore - 560074, Karnataka, India; 2 Department of General Medicine, Akash Institute of Medical Sciences and Research Centre, Bangalore - 562110, Karnataka, India; 3 Department of General Medicine, Akash Institute of Medical Sciences and Research Centre, Bangalore - 562110, Karnataka, India; 4 Department of Biochemistry, Akash Institute of Medical Sciences and Research Centre, Bangalore - 562110, Karnataka, India

**Keywords:** Coronary Artery Disease, Hypertension, T2DM and Interleukin 6

## Abstract

Type 2 Diabetes Mellitus (T2DM) patients with coronary artery disease (CAD) coupled with hypertension (HTN) are leading cause of morbidity and mortality. Association of Interleukin 6 and uric acid levels can be used for early detection of CAD in patients with T2DM. This cross sectional study included 120 study participants, were sub grouped into three Group 1: controls (n=30), Group 2: CAD, HTN without T2DM (n=30) and Group 3: CAD, HTN with T2DM (n=30). Basic biochemical, clinical and experimental parameters were analysed and recorded for all the study subjects. The statistical analysis was done by using the SPSS 20.0. Individuals with CAD, HTN with T2DM had higher BMI, weight, SBP, and DBP levels than patients CAD, HTN without diabetes and controls. In CAD, HTN with T2DM study participants has been found to have higher serum levels of inflammatory cytokines like IL-6 and uric acids when compared to CAD, HTN without T2DM and controls. IL6 was significantly positive association with uric acid and negative association with age and HDL. These findings demonstrated a statistically significant positive connection between IL-6, and uric acid concentrations. The identified elevated levels of inflammatory cytokines and uric acids in type 2 diabetic and hypertensive CAD patients may aid in the identification of patients at higher risk.

## Background:

One of the most prevalent metabolic illnesses, Type 2 Diabetes Mellitus (T2DM), is brought on by a confluence of two main factors: improper insulin secretion by pancreatic beta-cells and improper insulin response in insulin-sensitive organs [1]. The molecular mechanisms involved in the synthesis, release, and detection of insulin are carefully regulated activities because they are necessary for maintaining glucose homeostasis [2]. A metabolic imbalance that is the cause of the disease's onset can result from flaws in any of the mechanisms involved in these processes. As the illness worsens,insulin secretion becomes unable to keep glucose levels in balance, leading to hyperglycemia [3]. Obesity or having a greater body fat percentage, primarily in the abdominal area, is the main characteristics of T2DM patients. Through a variety of inflammatory mechanisms, including increased free fatty acid (FFA) release and adipokine dysregulation, adipose tissue promotes IR in this situation [4]. The prevalence and incidence of type 2 diabetes (T2DM) have quadrupled as a result of population ageing, sedentary lifestyles, high-calorie diets, and global obesity rates [5].This over time leads to damage the heart,vasculature, eyes, kidneys and nerves. Alarming statistics from epidemiological data indicate a worrying projected future for T2DM. The International Diabetes Federation (IDF) estimates that 463 million adults between the ages of 20 and 79 had diabetes in 2019, and that number is expected to reach 700 million by the year 2045 [6].In 2019, diabetes caused 4.2 million deaths worldwide. At least 720 billion USD in medical expenses were related to diabetes in 2019. Additionally, since 1 in 3 diabetics-or 232 million people-had their diabetes misdiagnosed, the true disease burden of T2DM is probably underrepresented [7]. Patients with either type 2 diabetes are at a higher risk of developing a number of cardiovascular diseases, including coronary heart disease, stroke, peripheral arterial disease, cardiomyopathy, and congestive heart failure, according to abundant data [8]. The primary contributors to morbidity and mortality from diabetes are now cardiovascular problems. Cardiovascular disease (CVD) in diabetic patients already has a significant and growing influence on public health. In clinical settings, diabetes mellitus (DM) and cardiovascular disease (CVD) frequently coexist; nevertheless, the pathophysiology of this comorbid condition may be somewhat unclear given the dispersed and growing body of scientific knowledge, particularly in the last ten years [9]. Diabetes has a negative impact on every aspect of the cardiovascular system, including the kidneys, heart, major arteries, and microvasculature [10]. Due to its rising prevalence in our society, diabetes is now considered to be one of the main risk factors for CVD, on par with hypertension, cholesterol problems, and cigarette smoking. It is a particularly significant risk factor for women and the ageing population in general. Proinflammatory mediators and/or cytokines, particularly interleukin-1 beta (IL-1), interleukin-6 (IL-6), tumor necrosis factor alpha (TNF-alpha), and various other IL-1-dependent pro-inflammatory cytokines and chemokines, are the primary causes of insulin resistance by inducing different inflammatory responses. Among the key inflammatory cells is interleukin-6 (IL-6), which primarily contributes to various inflammatory processes by regulating cell proliferation, migration, differentiation, and death. The etiology of atherosclerosis, the primary cause of coronary artery disease, is heavily reliant on IL-6 signaling (CAD) [11-12]. Multiple factors, including oxidative stress and vascular damage, can increase IL-6 secretion. It is widely known that high levels of circulating IL-6 are associated with a higher risk of cardiovascular events.Existing studies inconsistency the present study aimed to determine the association of interleukin 6 and uric acid levels in patients with CAD, HTN with and without T2DM.

## Materials and Methods:

This cross sectional prospective observational study was included 60 patients diagnosed with CAD with and without T2DM as per American Diabetes Association (ADA) criteria during the period of March 2018 to February 2021 and attending Endocrinology and metabolism and Nephrology OPD at Akash Institute of Medical Sciences, Tirupati, was included into the study. Thirty age, gender and BMI matched healthy controls were included in Group 1. The patients were divided into two groups of thirty for each group CAD, HTN without T2DM and CAD, HTN with T2DM. Informed consent of the subjects was taken after explaining the study in their vernacular language. The study was conducted after obtaining approvals by the Institutional thesis protocol approval committee and Institutional ethics committee.

## Criteria of the study:

## Inclusion criteria:

The patients diagnosed with CAD, HTN with and without T2DM. CAD diagnosed by angiogram. All the study subjects' age should be 30 to 70 years.

## Exclusion criteria:

Patients with pregnant, lactating women, smoking, alcoholism, autoimmune disorders, liver diseases, kidney diseases, other types of diabetes mellitus, thyroid diseases, congenital heart problems, other types of heart diseases, and people who were unwilling to participate in the study were excluded from the study.

## Collection of blood sample:

Ten mL of fasting and 3 mL of postprandial venous blood samples were drawn from the cubical fossa of each participant. After collection of blood samples centrifuged at 3000 RPM for 10 minutes, plasma and serum was separated and transferred into properly labeled aliquots stored at -800 C until biochemical analysis was done.

## Methods:

Plasma samples were used for the determination of fasting and postprandial blood sugars, whole blood used for estimation of glycosylated hemoglobin (HbA1c) and serum sample was used for to analyze biochemical and experimental parameters by using laboratory standard methods. Interleukin 6 was measured by using Enzyme Linked Immuno Sorbent Assay (ELISA).

## Statistical analysis:

The normal distribution of data is checked using Kolmogorov Smirnov test. All the characters descriptively summarized. The mean and standard deviation about the arithmetic mean were used. Dependent variables should be normally distributed, for comparison of variables by using analysis of variance (ANOVA). Pearson correlation analysis was used for correlation of BMI, uric acid and IL6 with other variables. The Data was compiled in Microsoft excel spread sheets and analyzed using SPSS for windows version 21.0. A p value < 0.05 was considered statistically significant.

## Results:

According to Table 1(see PDF), patients with hypertension CAD and type 2 diabetes mellitus had mean ages that were statistically significantly different from those of controls (55.83 5.47 vs. 55.42 9.02 vs. 54.75 8.96, respectively; P = 0.0001**). When compared to healthy controls (23.603.20, P=0.0001**), there were very significant differences in BMI between patients with and without type 2 diabetes mellitus and hypertension (32.934.31, 34.704.76). In addition, we found that the differences in systolic and diastolic blood pressure between hypertension CAD patients with and without type 2 diabetes mellitus (153.394.01, 174.297.65, and 94.293.62, respectively) and healthy controls (126.124.79 and 77.953.01, respectively, were statistically significant (P=0.0001**).

## Comparison of biochemical and experimental parameters among the study subjects:

Table 1(see PDF) presents the findings from an analysis of the key biochemical and clinical variables related to CAD disorders. When group 3 and group 2 were compared to group 1, there were significantly higher levels of FBS (200.9218.45, 89.6810.23, 80.208.24), PPBS (374.555.77, 124.658.43, 129.6513.13), HbA1c (13.336.08, 9.245.49, 9.245.58), total cholesterol (358.1555.00, 284.3212. This implied that people with diabetes and hypertension were more likely to develop coronary artery disease. In order to further investigate, it was discovered that group 2 subjects had significantly higher TGL and VLDL concentrations than group 3 and group 1 subjects. The P values were found to be highly statistically significant (P= 0.0001**). BMI, Uric acid, interleukin-6, and other indices were evaluated to further explore the links and the results are displayed in Table 2(see PDF). As stated, BMI, uric acid and interleukin-6 were significantly negatively correlated with HDL (r = -0.875, -0.549 and -0.748) respectively P value is 0.0001**and positively correlated with FBS, PPBS, HbA1c, TC, TGL, and LDL, respectively. As opposed to this, there was no association between uric acid and TGL (r= 0.135, P=0.142). Figure 1(see PDF) shows the interleukin 6 concentrations in two groups of study subjects. The patients with CAD, HTN and T2DM patients shown a significantly increased when compared to CAD, HTN without T2DM and controls. Figure 2(see PDF) shows the uric acid concentrations in two groups of study subjects. The patients with CAD, HTN and T2DM patients shown a significantly increased when compared to CAD, HTN without T2DM and controls. Figure 3(see PDF) shows the scatter plots of IL6 and BMI among the study subjects, there was a significantly positive association between IL6 and BMI. Figure 4(see PDF) shows the scatter plots of uric acid and BMI among the study subjects, there was a significantly positive association between uric acid and BMI.

## Discussion:

Heart artery atherosclerosis is a defining feature of the condition known as CAD (blood vessels responsible for supplying the heart with blood). The main cause of this disease is the slowly narrowingof the blood vessel walls that carry blood to the heart, caused by lipid plaques that induce ischemia (occlusion or narrowing of the blood vessel), which results in a lack of nutrients and oxygen to the heart muscle [13-14]. T2DM is a significant contributor to the likelihood of developing CAD.Studies have shown that prolonged chronic hyperglycemia is mostly or substantially responsible for cardiovascular complexity[15]. It is made clear that the increased incidence of CAD in individuals with type 2 diabetes cannot be explained solely by changes in traditional risk factors such elevated blood pressure and aberrant cholesterol levels [16]. Additionally, one of the factors that contribute to the aetiology of CAD, insulin resistance (IR), and T2DM is persistent subclinical vascular inflammation. It has been demonstrated that the markers of subclinical inflammation, such as IL-6, are significant independent predictors of T2DM and CAD risk [17]. In our study also observed significantly elevated levels of IL6 in both the groups of CAD, HTN with and without T2DM patients when compared to healthy controls. There was a significantly positive association between IL6 and FBS, PPBS, Lipid Profile and uric acid, negative association between IL6 and HDL among the study subjects. Another recent study also reported significantly elevated levels of IL6 positively correlated with diabetic profile and uric acid [18]. These findings suggest that individuals at risk for future myocardial infarction who appear healthy have considerably increased baseline levels of the inflammatory cytokine IL-6. Analyses that corrected for baseline differences in total cholesterol, HDL cholesterol,body mass index, blood pressure, diabetes, etc. did not change the link between IL-6 level and risk in patients with T2DM [19].We observed a relationship between elevated serum uric acid levels and an increased risk of developing CAD in patients with type 2 diabetes due to the significant elevation of serum uric acid levels in patients with CAD, HTN with/without T2DM. These relationships maintained in both genders and were unaffected by other type 2 diabetes risk variables such as age, BMI, alcohol intake, smoking, degree of physical activity, hypertension, and blood sugar, cholesterol, creatinine, and triglyceride levels.Overall, these results offer prospective proof that those with greater serum uric acid, including younger adults, are more likely to develop CAD in patients with type 2 diabetes [20-21].Hyperuricemia in type 2 diabetic individuals appears to increase their chance of acquiring diabetic complications, particularly renal and cardiovascular disease [22].Hyperuricemia appears to be linked to the insulin-resistance syndrome, decreased glucose tolerance, insufficient metabolic regulation and increased blood vessel filtration. Even though it is one of the most important antioxidants in the body, uric acid can cause oxidative stress in many different types of cells, including vascular smooth muscle cells, which can then accelerate the development of cardiovascular disease in patients with T2DM [23-24]. Significant IL-6 and uric acid production in both the groups of CAD, HTN with and without T2DM when compared to healthy controls might be used for early men interventions of myocardial infarction in the future.

## Conclusion:

This study concludes significantly elevated levels of interleukin 6 and uric acid in CAD, HTN with and without T2DM patients might be useful for to predict early signs of myocardial infarction.

## References

[1] Galicia-Garcia U (2020). Int J Mol Sci..

[2] Javeed N (2018). Physiology (Bethesda)..

[3] Brunton S (2016). J Fam Pract..

[4] Gurung M (2020). EBioMedicine..

[5] Guthrie RA (2004). Crit Care Nurs Q..

[6] Sampath Kumar A (2019). Ann Phys Rehabil Med..

[7] Valaiyapathi B (2020). Future Cardiol..

[8] Henning RJ (2018). Future Cardiol..

[9] Akbari M (2018). Inflammopharmacology..

[10] Giraldez MD (2021). Nat Rev Gastroenterol Hepatol..

[11] SjÖholm A (2006). Diabetes Metab Res Rev..

[12] Xiang J (2020). Front Endocrinol (Lausanne)..

[13] Akbari M (2018). Inflammopharmacology..

[14] Siewko K (2019). Biomed Res Int..

[15] Zhang X (2021). J Diabetes Res..

[16] Dai CY (2008). Diabetes Care..

[17] Lippi G (2008). Diabetes Care..

[18] Katsiki N (2021). Curr Pharm Des..

[19] Chang Z (2021). Prim Care Diabetes..

[20] Katsiki N (2013). Curr Pharm Des..

[21] Su H (2021). Prim Care Diabetes..

[22] Galváo AIR (2021). Cad Saude Publica..

[23] Danesh J (2008). PLoS Med..

[24] Bradham WS (2014). Inflammation..

